# Supplementary material for: Heme Oxygenase-1 and Blood Bilirubin Are Gradually Activated by Oral D-Glyceric Acid
Source: Antioxidants (Basel). 2022 Nov 23;11(12):2319. doi: 10.3390/antiox11122319 (PMC9774343; doi:10.3390/antiox11122319)
Supplement: Supplementary file 1 [file antioxidants-11-02319-s001.zip › supplementary S1 ROS in hepatocytes.pdf]

Supplementary **S1. Human primary hepatocyte ROS study model and results**

Selection of the 50-60-year-old ex vivo donors: persons with normal liver function. Preferably no or only moderate ethanol consumption, no tobacco, and BMI in the range of [25, 31]. Cause of death cardiovascular accident (CVA) or related, i.e., no serious problems with liver function.

In the study model mild metabolic stress was induced by the change of the cultivation media 1.5 or 2 h before the ROS measurement, i.e., mild metabolic stress arises from new nutrition. Recommended interval for the change of the cultivation medium was 24 h.

Figure S1 A-C. ROS generation of hepatocytes from CDP (**A**), JGM (**B**) and DOO (**C**).

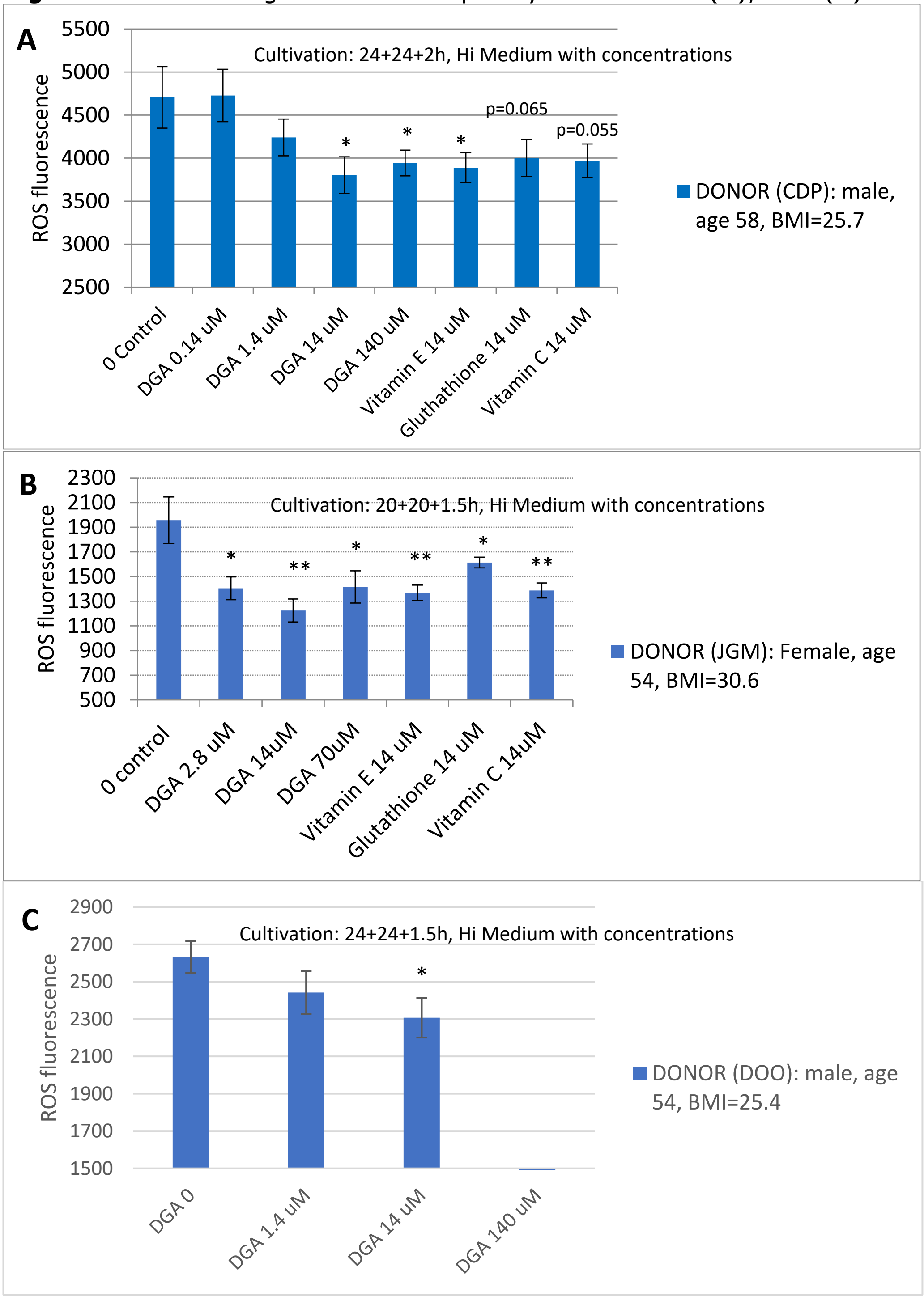

The dose of vehicle, DGA, and the antioxidant was always administered with the new cultivation medium. Furthermore, the hepatocytes were primed for two days with the vehicle, DGA, or antioxidant before the last dose and subsequent ROS measurement 1.5 or 2 h thereafter (see Figs. A and C). With the donor JGM (Fig. B), we exceptionally tried 20 h cycle in the change of the cultivation medium. In this test the ROS reducing effect of DGA was statistically very significant (Fig. B).

Results

For all donors ROS generation declined statistically significantly with DGA concentration of 14 umol/L (µM). In equimolar 14 µM comparison with vitamins C and E and glutathione the ROS scavenging effect of DGA was at least as good or even somewhat superior to mentioned antioxidants (Figs. A and B).

Cell viability was detected with LDH. No major changes in cell viability were observed except for vitamin E (Trolox). For Trolox cell viability during the cultivation period was reduced, and thus the ROS generation in Figs. A and B may underestimate real ROS generation per viable cell in the case of vitamin E because the number of viable cells was reduced when measuring ROS.

Materials and methods for hepatocyte studies

Primary human hepatocytes were purchased from Celsis In Vitro Technologies (1450 South Rolling Road Baltimore, MD 21227, USA). Primary hepatocytes from altogether 3 donors aged 57 (DOO, male), 58 (CDP, male), and 54 (JGM, female) were used. According to the information provided by the Celsis, hepatocytes from each donor should have at least 70 % viability and more than 5 million viable cells. The medium for the culture of hepatocytes was provided by Celsis. They were InVitroGRO CP (for plating) medium (Z99029) and InVitroGRO HI (for incubation) medium (Z99009). Antibiotics (Torpedo Antibiotics Mix, Z990007) were also from Celsis.

Thawing, plating, and culture of cells were carried out according to the instruction provided by the Celsis In Vitro Technologies. The other reagents for experiments were D(-) fructose (Sigma-Aldrich, F0127), D(+) -glucose (Sigma-Aldrich G7528), Dulbecco's phosphate buffered saline (DPBS) (Lonza, BE17-512F), absolute ethanol (ProLab 20821.365) and foetal bovine serum (Thermo Fisher, SV30160). For the cell culture, BD BioCoat™ Collagen I Coated 96-well Black/Clear Plates (354649) were used. Other plastic ware used in this study was purchased from Sarstedt Ltd (Leicester LE4 1AW, UK). Cells were cultured in a cell culture incubator (Sanyo MCO-18AIC) at an atmosphere of 37°C and 5% CO2.
For the measurement of cell viability after treatment of test compound, CytoTox-One Homogeneous Membrane Integrity Assay kit (Promega, G7891) was used. The CytoTox-One Assay is a rapid fluorescent measure of the release of lactate dehydrogenase (LDH) from cells with a damaged membrane. The number of viable cells correlates to the fluorescence intensity determined by a fluorescence plate reader (Hidex Chameleon V multiplate reader, Hidex Oy, Turku, Finland) with excitation 544 nm and emission 590 nm. For the measurement of cellular reactive oxygen species (ROS), DCFDA Cellular ROS Detection Assay Kit from Abcam (ab113851) was used. Reactive oxygen species (ROS) assay kit (ab113851) uses the cell permeant reagent 2',7'-dichlorofluorescein diacetate (DCFDA), a fluorogenic dye that measures hydroxyl, peroxyl and other ROS activity with the cell. The activity of ROS was measured by a fluorescence plate reader (Hidex) with excitation/emission wavelengths of 485 nm/535 nm. This study was conducted in accordance with the relevant standard operating procedures (SOPs) in BioSiteHisto Oy.

Cell culture

The culture of primary hepatocytes was carried out based on the instructions provided by Celsis. After thawing, the cells were suspended in InVitroGRO CP medium. Thereafter the viability of cells was determined using the Trypan Blue exclusion method. Then the concentration of cells was adjusted using InVitroGRO CP medium, so about 30000 – 35000 cells / 100 µl / well can be seeded in collagen I coated 96 well plate (BD, 734-0248).
After seeding, the cells were cultured overnight in the cell culture incubator (Sanyo MCO 18) at atmosphere of 95% air and 5% CO2 at 37°C. Thereafter, the culture medium for the cells was changed to InVitroGRO Hi

(Hi Medium) containing test compound, D-glyceric acid calcium salt dehydrate (DGA), at the selected concentrations. DGA (molecular weight=106 g/mol) was also tested in equimolar doses against other substances with known antioxidative properties, i.e. vitamin E (trolox, T3251 Sigma, molecular weight=431 g/mol), glutathione (G6013 Sigma, molecular weight=307 g/mol), vitamin C (L-ascorbic acid, A4544 Sigma, molecular weight=176 g/mol) and morin dehydrate (M4008 Sigma, molecular weight=302 g/mol).
After 24 h (Figs. A and C)or 20 h (Fig. B) incubation medium was renewed. Second renewal of the incubation medium occurred at 48 h or 40 h. ROS was measured after 1.5-2 hours after last change of the medium, i.e. in a situation with moderate metabolic stress.

LDH measurement

The measurement of LDH from medium (leaked LDH) and from cell and medium (total LDH) was carried out according to the instruction provided by the Promega (G7891). The plate was incubated at an incubator to achieve a temperature of 22°C. Thereafter, equal volume of CytoTox-ONE Reagent to cell culture medium (100 µl) was added to each well and mix for 30 seconds. Then they were incubated for 10 min at 22°C, and then 50 µl of Stop Solution was added to each well. After gentle mixing, the fluorescence signal was measured at an excitation wavelength of 560 nm and emission wavelength of 590 nm using the Hidex Chameleon V multiplate reader (Hidex Oy, Turku, Finland).
For the total LDH measurement, all the steps are same except 2 µl of Lysis Solution will be added to the each well to lyse the cells before CytoTox-ONE Reagents will be added.

Cellular ROS detection

The measurement of cellular reactive oxygen species (ROS) was carried out according to the instruction of Abcam (ab113851). At the end of treatment time point, culture medium was taken away for leaked LDH measurement and cells were washed with 100 µl / well PBS once. Thereafter 100 µl / well of DCDA mix was added and incubated for 45 min at 37 °C in the dark. Then cells were washed once with buffer solution. The fluorescence signal was measured at an excitation wavelength of 485 nm and emission wavelength of 535 nm using the Hidex Chameleon V multiplate reader (Hidex Oy, Turku, Finland).

Calculation and interpretation of results

For the LDH and ROS assays, individual values of each measurement were stored and average value of fluorescence signals of various repeats of each treatment at studied time points were calculated and compared. All ROS calculations possessed 6 repeats for all measurement point. Viability (LDH) measurements contained 4-6 repeats depending on the study. Detailed study plans and plate setups etc. can be obtained from corresponding author.

1. Tsuchihashi, S.-I.; Livhits, M.; Zhai, Y.; Busuttil, R.W.; Araujo, J.A.; Kupiec-Weglinski, J.W. Basal rather than induced heme oxygenase-1 levels are crucial in the antioxidant cytoprotection. *J. Immunol.* **2006**, *177*, 4749–4757. https://doi.org/10.4049/jimmunol.177.7.4749.
2. Nightingale Health UK Biobank Initiative; Julkunen, H.; Cichońska, A.; Slagboom, P.E.; Würtz, P. Blood biomarker score identifies individuals at high risk for severe COVID-19 a decade prior to diagnosis metabolic profiling of 105000 adults in the UK Biobank. *Front. Immunol.* **2021**, *12*, 809937. https://doi.org/10.1101/2020.07.02.20143685.
