# Supplementary material for: Heme Oxygenase-1 and Blood Bilirubin Are Gradually Activated by Oral D-Glyceric Acid
Source: Antioxidants (Basel). 2022 Nov 23;11(12):2319. doi: 10.3390/antiox11122319 (PMC9774343; doi:10.3390/antiox11122319)
Supplement: Supplementary file 1 [file antioxidants-11-02319-s001.zip › supplementary S2 ROS in astrocytes final.pdf]

Supplementary S2. Astrocyte ROS study, study outline, main results and materials and methods

Experimental Design: Evaluation of the efficacy of DGA on cell viability in response to oxidative stress primary optic nerve astrocytes and the production of reactive oxygen species (ROS)

SUMMARY The objective of this study was to assess the efficacy of DGA in scavenging the acute production of reactive oxygen species (ROS) (15 min – 240 min) in response to normal metabolism (change of the media) and additionally in response to oxidative stress caused by bolus addition of tBHP (tert-Butyl hydroperoxide) in primary optic nerve astrocytes. As an important part of the study the cell viability was estimated 6 hours after tBHP addition by two methods, i.e., LDH and MTT assays.

Study outline: Primary optical head nerve astrocytes were obtained from 4-week-old male Wistar rats and where cultured to obtained sufficient cells for the project. Cells were plated at 5000 cells per well and allowed to adhere for 24 hrs prior to the addition of compounds for 48 hrs (replenished every 24 hrs). After 24 hrs the compounds were removed and added for 1 hr, prior to the addition of tBHP at 85 µM as detailed below after the results. ROS detection using CellROX green was performed over a time course of 4 hrs. MTT and LDH assay (release and total LDH) were performed after 6 hrs.

The overall objective of this study was to analyze whether 14 µM DGA possesses the ability to protect primary optical nerve head astrocytes in the presence of low levels of ROS related to mild metabolic stress, and whether it abrogates an increase of intracellular ROS upon the bolus addition of tBHP. The concentration of 14 µM DGA was chosen because in earlier dose finding studies with human primary hepatocytes it was found out to be efficient in ROS scavenging(see Appendix 1). Also, the 48 h (24h+24h) priming was identical to the hepatocyte studies.

Results

Figure S2 14µM DGA concentration against 0-control with or without tBHP. All cells primed for 48 h with either vehicle (0-controls) or 14 µM DGA. (In Figs. A and B uM = µM = µmol/L.)**(A)** ROS generation, 2 h after new media and 1 h after tBHP addition**(B)** ROS generation, 1 h 15 min after new media and 15 min after tBHP addition.

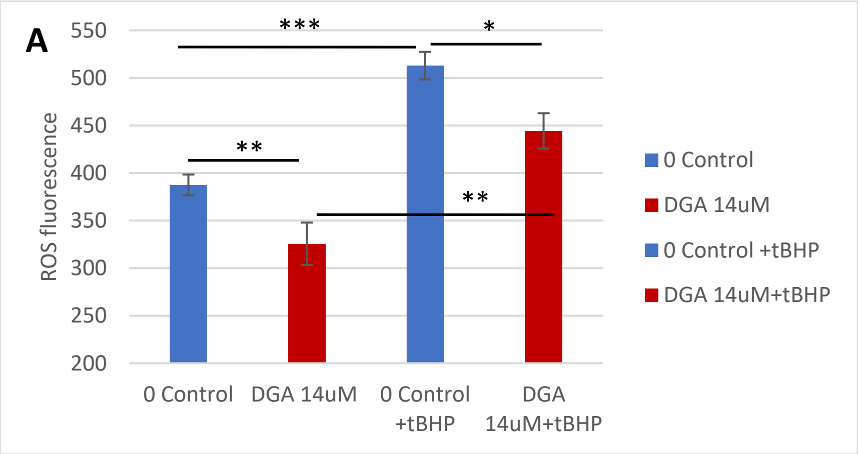

‘) One clear outlier observation was deducted from 14 µM DGA without tBHP and 0-control without tBHP.

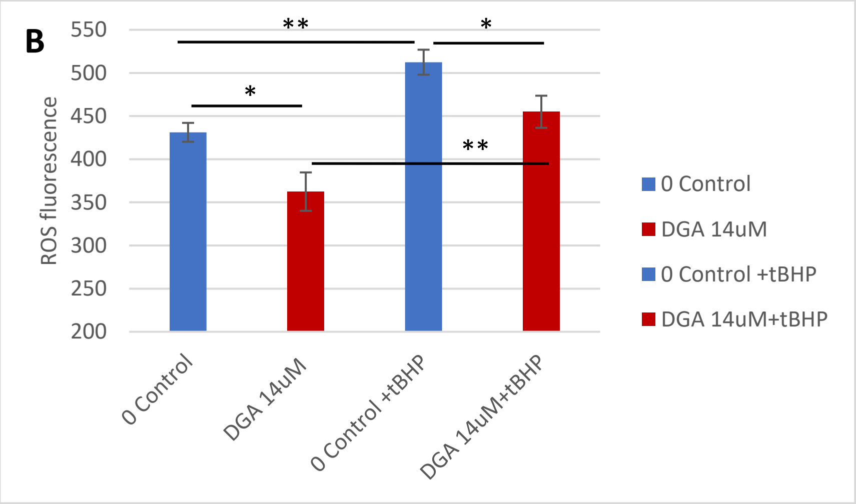

‘) One clear outlier observation was deducted from 14 µM DGA without tBHP and 0-control without tBHP.

The main findings where:

1. Oxidative stress induced by normal culturing and related change of media 1-2 hours earlier was statistically significantly decreased in the presence of 14 µM DGA against the 0-control (N=5).
    a. The ROS scavenging effect with primary rat astrocytes was very similar to the results obtained with primary human hepatocytes earlier. Main effective concentration was the same 14 µM DGA in both but also other tested DGA concentrations reduced ROS generation.
2. Oxidative stress induced by tBHP was statistically significantly decreased in the presence of 14 µM DGA against control (N=6).

Other related findings:

3. DGA did not cause significant cell viability changes compared to the culturing with the 0-control without tBHP.
4. The selected dose of 85 µM tBHP increased ROS by optimal 20% and that effect was statistically very significant. It did not cause acute cell death but towards the end of altogether 6-hour follow-up time there was a subtle 20% decrease in cell viability.
5. It was observed that when the cell viability in “14 µM DGA with 85 µM tBHP” group was compared to “0-control with 85 µM tBHP” group, the DGA treatment gave some protection against tBHP although no full protection against cell death was seen.
    a. This was consistent with the observed ROS reductions in the DGA groups.
    b. Viability effect was a secondary issue in the study.

Discussion (references are in the main article)

The protective mechanism of DGA is not fully known but based on the findings in this article it may be related to the activation of HO-1 pathway [25] and related generation of biliverdin / bilirubin within studied astrocytes [5,6]. Because DGA administration seems to activate mitochondrial energy metabolism in vivo [27] it is also possible that reverse electron transport (RET) in the OXPHOS is downregulated. RET may possess an important role in cellular ROS generation [46]. There exists also the possibility that DGA forms similar cytosolic antioxidant loop with HPA as bilirubin forms with biliverdin [32]. Further studies are needed.

References:

[5] T. W. Sedlak, M. Saleh, D. S. Higginson, B. D. Paul, K. R. Juluri, and S. H. Snyder, Bilirubin and glutathione have complementary antioxidant and cytoprotective roles, *Proc Natl Acad Sci U S A*, vol. 106, no. 13, pp. 5171–5176, 2009, doi: 10.1073/pnas.0813132106.

[6] S. Jayanti, R. Moretti, C. Tiribelli, and S. Gazzin, Bilirubin: A promising therapy for parkinson's disease, *International Journal of Molecular Sciences*, vol. 22, no. 12. MDPI, Jun. 02, 2021. doi: 10.3390/ijms22126223.

[25] S. Tsuchihashi, M. Livhits, Y. Zhai, R. W. Busuttil, J. A. Araujo, and J. W. Kupiec-Weglinski, Basal Rather Than Induced Heme Oxygenase-1 Levels Are Crucial in the Antioxidant Cytoprotection, *The Journal of Immunology*, vol. 177, no. 7, pp. 4749–4757, 2006, doi: 10.4049/jimmunol.177.7.4749.

[27] O. P. Hirvonen, H. Kyröläinen, M. Lehti, and H. Kainulainen, Randomized Trial: D-Glyceric Acid Activates Mitochondrial Metabolism in 50–60-Year-Old Healthy Humans, *Frontiers in Aging*, vol. 2, Oct. 2021, doi: 10.3389/fragi.2021.752636.

[32] P. Hirvonen, P. C. J. Eriksson, and R. Kaksonen, Method for enhancing energy production and metabolism in cells, WO2015036656A3, 2014

[46] F. Scialò, D. J. Fernández-Ayala, and A. Sanz, Role of mitochondrial reverse electron transport in ROS signaling: Potential roles in health and disease, *Front Physiol*, vol. 8, no. JUN, pp. 1–7, 2017, doi: 10.3389/fphys.2017.00428.

2. Materials and Methods related to ROS Measurements from Astrocytes

2.1. Cell Culture

The primary optical nerve astrocytes have been isolated from 2 individual rats grown and frozen at different passages. The astrocytes are available at different passages. Early passages were used.

These cells were thaw and grown in culture for 1 passage prior to the start of the experiment. The cell culture medium was DMEM high glucose, 20% FBS, 5 U potassium penicillin and 5 µg/mL streptomycin sulfate). 0.25% Trypsin-EDTA was used as the detachment method. Aseptic cell culture techniques were used, and the cells were grown in 5% CO2, 90% humidity, 37^0C cell culture incubator.

For the ROS measurements, the preloading of the dye and subsequent microplate measurements, were done in phenol free DMEM with 20% FBS.

2.2. Cell Seeding in 96 well plates

Primary cultures must have no more than 10% cell death prior to seeding as determined by manual counting using Erythrosin B on a haemocytometer.
The cells will be seeded in 96 well plates at a seeding density of 5000 cells per well in 100 ul of media. For the MTT, LDH and RNA analysis Greiner tissue cultures plates will be used. For the ROS analysis Greiner optical clear black wells will be used. These are certified DNase and RNase free plastic wear.
The cells will be allowed to adhere overnight prior to treatment.
Total number of cells needed for the whole study: $2x10^6$ cells, seeded at 5000 cells per well.

2.3. Schematic representation of the Experiment

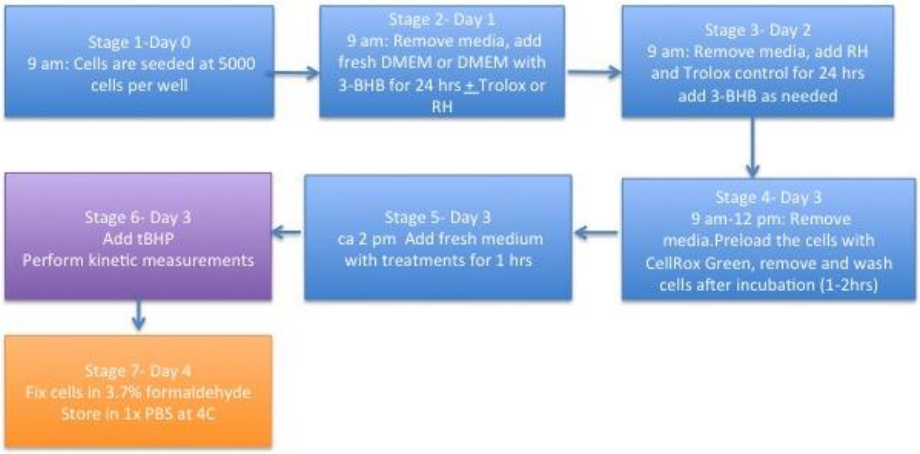

At each stage the condition of the cells were visually assessed using a light microscopy.

Stage 1-3 are as described under MTT and LDH assays. Use complete DMEM Phenol Free medium.

Stage 4: The media will be removed and saved and the cells loaded with CellRox Green in the presence of complete phenol free DMEM and allowed to incubate at 37°C in the cell culture incubator.
Stage 5: Following loading of the dye, the cells will be washed with DMEM phenol free medium to remove excess exogenous dye.
The cell culture media from step 4 and added back and toped-up with complete phenol free DMEM containing DGA and Trolox for 1 hr.

Stage 6: The bolus addition of tBHP will be added at less than 1/10 volume of the media and at the concentration that gives 25% killing by 6 hrs (dilution to be determined after the dose response curve is generated and after discussion with Replicon). The compound is not removed from the medium prior to addition of the tBHP. This is 0 minute.

Stage 7: The cells will be read in a fluorescent microplate reader at 15 and 30 minutes, 1, 2,3 and 4hr.

2.4. The Plate Design for the ROS Analysis *(RH = DGA calcium salt dehydrate)*

The plate was designed according to the study proposal and 1 plate with 6 replicates were used. The outer wells were not used due to edge effects. Greiner tissue culture optical clear black well plates were used.

One identical plate => 6 repeats for all. Treatments 24 h + 24 h + 4 h. Add CellRox Dye roughly at 24h+23h. Add "YY tBHP" only at 24+24h.

		Controls	RH Treatment	RH Treatment	Controls	RH Treatment	RH Treatment	RH Treatment	Trolox positive control	Controls	RH Treatment	
	1	2	3	4	5	6	7	8	9	10	11	12
	CellROX Green preloaded	XX hr / XX = time point when 25% cell death has been reached										
		tBHP 0 0 control	tBHP 0 RH 14 µM	tBHP 0 RH 70 µM	tBHP YY 0 control	tBHP YY RH 14 µM	tBHP YY RH 70 µM	tBHP YY RH 140 µM	tBHP YY Trolox 70 µM	tBHP YY BHB 125 uM	tBHP YY / bHB 0,125 RH 70 µM	
A	water / medium only	water / medium only	water / medium only	water / medium only	water / medium only	water / medium only	water / medium only	water / medium only	water / medium only	water / medium only	water / medium only	water / medium only
B	no cells / background	0 µg/ml	2 µg/ml	10 µg/ml	0 µg/ml	2 µg/ml	10 µg/ml	20 µg/ml	Trolox 70 µM	BHB 125 uM	10 µg/ml	no cells / background
C	no cells / background	0 µg/ml	2 µg/ml	10 µg/ml	0 µg/ml	2 µg/ml	10 µg/ml	20 µg/ml	Trolox 70 µM	BHB 125 uM	10 µg/ml	no cells / background
D	no cells / background	0 µg/ml	2 µg/ml	10 µg/ml	0 µg/ml	2 µg/ml	10 µg/ml	20 µg/ml	Trolox 70 µM	BHB 125 uM	10 µg/ml	no cells / background
E	no cells / background	0 µg/ml	2 µg/ml	10 µg/ml	0 µg/ml	2 µg/ml	10 µg/ml	20 µg/ml	Trolox 70 µM	BHB 125 uM	10 µg/ml	no cells / background
F	no cells / background	0 µg/ml	2 µg/ml	10 µg/ml	0 µg/ml	2 µg/ml	10 µg/ml	20 µg/ml	Trolox 70 µM	BHB 125 uM	10 µg/ml	no cells / background
G	no cells / background	0 µg/ml	2 µg/ml	10 µg/ml	0 µg/ml	2 µg/ml	10 µg/ml	20 µg/ml	Trolox 70 µM	BHB 125 uM	10 µg/ml	no cells / background
H	water / medium only	water / medium only	water / medium only	water / medium only	water / medium only	water / medium only	water / medium only	water / medium only	water / medium only	water / medium only	water / medium only	water / medium only
	1	2	3	4	5	6	7	8	9	10	11	12

2.5. Outline of ROS measurements using a Fluorescent Microplate Reader

CellROX® Green Reagent (catalogue nr C10444, Invitrogen) is a novel fluorogenic probe for measuring ROS in live cells. This probe is a cell permeable dye which is weakly fluorescent while in a reduced state and exhibits bright green photostable fluorescence upon oxidation by reactive oxygen species (ROS) and subsequent binding to DNA, with absorption/emission maxima of ~ 485/520 nm. This reagent can be formaldehyde-fixed and its signal survives detergent treatment with Trition X-100, which makes it multiplexable with other compatible dyes e.g. DAPI, live-dead uptake and antibodies.

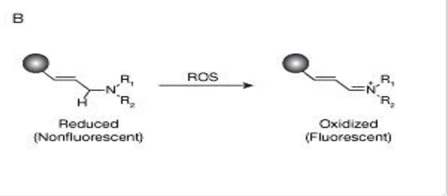

Mechanism of action. The reagent in cell permeable and produces an increase in fluorescence upon oxidation by ROS.

1. Cells are plated as detailed under section 2.1. cell seeding in optical clear black well plates.
2. Following attachment, the medium is removed and the cells are treated as in section 3.0 but in phenol free DMEM medium.
3. Medium is removed and kept in a sterile 96 well plate in the cell culture incubator.
4. The cells are preloaded with CellRox Green in phenol free media contain 20% FBS for 30 minutes. The cells are allowed to incubate at 37° in the cell culture incubator.
5. Following this the cells are washed 3 times with phenol free DMEM complete medium to removed access exogenous dye, the medium from point 3 is added back to the wells.
6. Fresh medium with or without DGA, Trolox and 3-BHB will be added for 1 hr.
7. The tBHP is then added and returned to the incubator and read at 15 min, 30 min, 1,2, 3 and 4 hr.
8. Read on the fluorescent microplate reader 530/30 band pass (BP) or similar using 3 readings per well.

2.6. Analysis

The averaged fluorescent measurement results. The difference of DGA compared to the 0-control with or without tBHP bolus addition.

2.7. Statistical Analysis for ROS Measurements

The plates contained 6 replicates. The data was averaged as means and S.D. determined. Student's t-test was used as statistical method but also non-parametric Wilcoxon-Mann-Whitney -test (also known as U-test or rank sum test) was used to ascertain statistically very significant deviation of 14 µM DGA from the 0-control in the case of added tBHP 15 min earlier.
Detailed study plan with Materials and methods also for the MTT and LDH viability measurements can be obtained from the corresponding author.
