# Supplementary figures and images for: Heme Oxygenase-1 and Blood Bilirubin Are Gradually Activated by Oral D-Glyceric Acid
Source: Antioxidants (Basel). 2022 Nov 23;11(12):2319. doi: 10.3390/antiox11122319 (PMC9774343; doi:10.3390/antiox11122319)

## CONSORT 2010 Flow Diagram

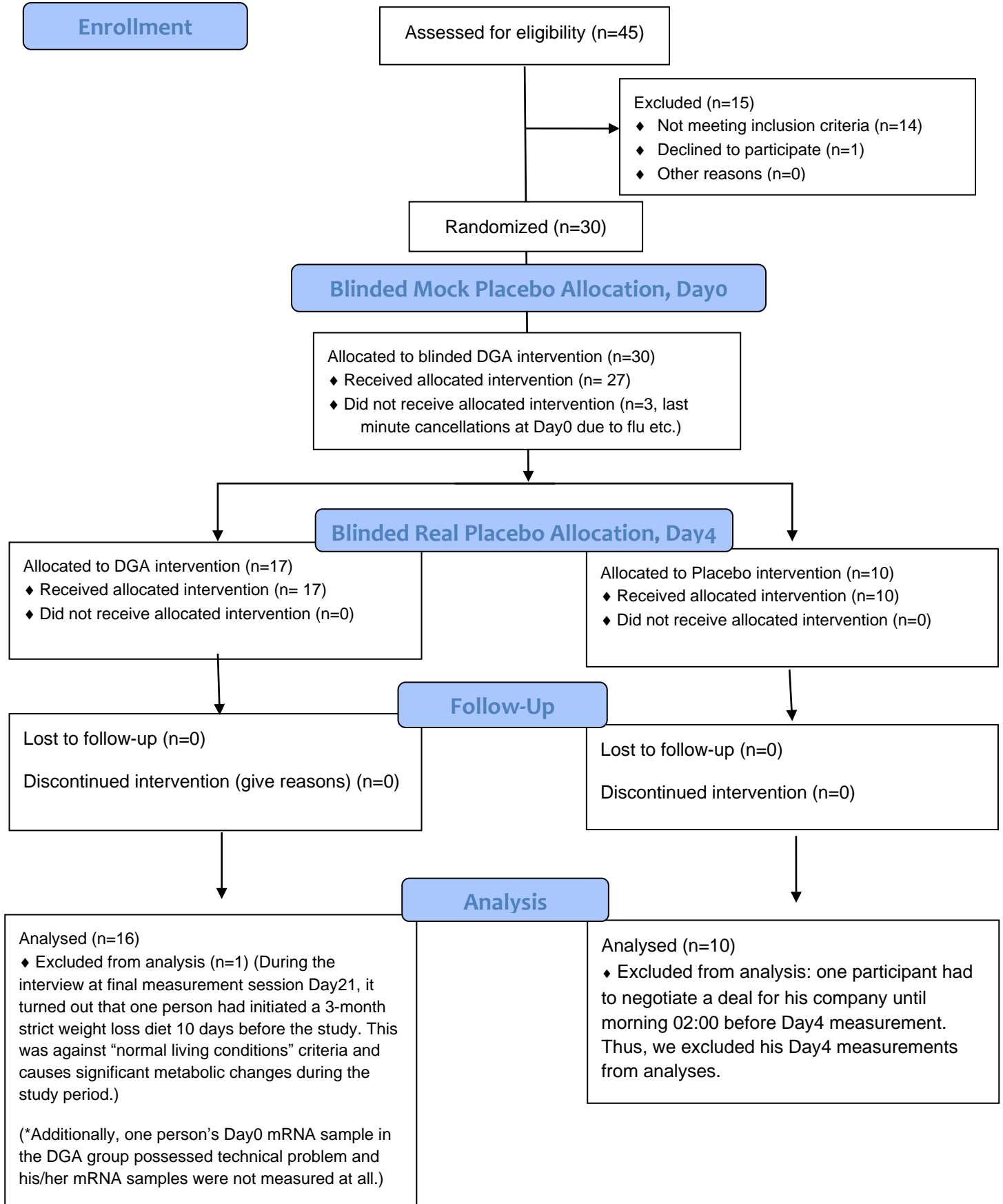

Supplement: Supplementary file 1 [file antioxidants-11-02319-s001.zip › CONSORT-Flow-Diagram.pdf]
